# The Medical, Surgical, and Hygienic Exhibition, 1900

**Published:** 1900-06-23

**Authors:** 


### THE MEDICAL, SURGICAL, AND HYGIENIC EXHIBITION, 1900.

(*Continued from page* 193.)

Last week we dealt with the drugs and surgical instruments which were displayed at this interesting exhibition. We now proceed to the foods and the various sanitary appliances.

The milk exhibits were evidence of the variety in which this all-important article of food can be produced in the highest purity and quality, and, by the adoption of every possible sanitary safeguard, under scientific supervision, rendered free from the suggestion of contamination. Messrs. Welford and Sons, of Elgin Avenue, Maida Vale, had a fine display, including asses' milk, of which they make a specialty. The Aylesbury Dairy Company, of St. Petersburgh Place, Bayswater, in an extensive exhibit, gave prominence to their Humanised Milk, which is esteemed the foremost of their products, and "Koumiss," which they prepare in three forms. The Friern Manor Farm (Limited), of 20, Farringdon Street, E.C., exhibited their Sterilised Milk, and also their Humanised Milk, which is prepared according to Dr. Gaertner's process. The Excel Milk Company, of 38, Vauxhall Bridge Road, S.W., showed specimens of their "Excel" sterilised milk in bottles. Mr. Henri Nestlé, 48, Cannon Street, E.C., gave a very attractive display of their condensed Swiss Milk, Viking Milk, and "Nestlé's Food." Condensed milk was also shown by the Anglo-Swiss Milk Company, 10, Mark Lane, E.C., whose "Milkmaid" brand is so widely favoured.

Messrs. Callard and Co., of 65, Regent Street, W., gave a most interesting exhibit of their foods for the diabetic, obese, rheumatic and gouty, and dyspeptic. Diabetic Marmalade, their latest production, deserves special mention. This firm have overcome the difficulty as to taste, and their foods are most palatable. Messrs. G. Van Abbott and Son, of 104, Wigmore Street, W., gave an extensive show of their high-class breads, biscuits, soups, &c. Worths Foods Syndicate (Limited), of Cheltenham, exhibited Cheltine diabetic and invalid foods, prominence being given to anæmic food. Messrs. J. G. Cox, of Gorgie Mills, Edinburgh, exhibited gelatine table jellies and flavouring essences; the jellies, which they invited the visitors to taste, were found to be delicately flavoured and pleasant eating. On the stall of Messrs. Cosenza and Co., of 95, Wigmore Street, W., were Maggi's Consommé, and Cross Star soups, which are held in high repute. The British Somatose Company (Limited), 165, Queen Victoria Street, E.C., had a large exhibit of their various preparations of Somatose. This is a nourishing and most convenient food for invalids, being in powder form, readily soluble in any fluid, and very easily digested. Liebig's Extract of Meat Company (Limited) exhibited their "Lemco," and "Peptarnis," and their stall received marked attention. Their preparations are so universally known and so highly valued as to need no recommendation. The Plasmon Syndicate (Limited), 56, Duke Street, Grosvenor Square, W., showed various preparations of Plasmon, including Plasmon Beef Extract, for which considerable merit is claimed as a nutrient as well as a stimulant. The Frame Food Company (Limited), Lombard Road, Battersea, S.W., exhibited their well-known preparations, which have attained such a high reputation for their nourishing properties. The stalls of Bovril (Limited) and Virol (Limited), Old Street, E.C., claimed much notice. Virol is a food for children and invalids, and is considered beneficial in cases of rickets, anæmia, &c. Messrs. Nelson Dale and Co., 14, Dowgate Hill, E.C., exhibited "Hipi," a mutton essence, which is an article of diet that should be much appreciated as an agreeable change from the customary beef tea. It makes excellent soup.

The Protene Company (Limited), 36, Welbeck Street, W., showed their milk proteid manufactures, including their latest productions, gingerbread and other fancy biscuits. These foods attracted the marked attention of the visitors. Messrs. Cadbury Bros. (Limited), Bournville, Birmingham, gave an interesting exhibit of their cocoa and chocolate, prominence being devoted to the cocoa as being the more important article of diet. Hovis bread, flour, biscuits, and infants' and invalid foods, which are in high public favour, were shown by the Hovis-Bread Flour Company (Limited), Macclesfield. Messrs. Broomfield and Co., of 83, Upper Thames Street, E.C., displayed their now familiar "Albene," a wholesome vegetable fat for cooking purposes and pastry. Albene forms a capital base for ointment. Cerebos (Limited), Newcastle-on-Tyne, exhibited their salt for table and culinary use. This excellent manufacture is a great improvement on common salt.

The Sanitas Company (Limited), of Bethnal Green, E., gave an extensive show of their "Sanitas" disinfecting fluids, oils, powders, and soaps, including Kingzett's patent preserved peroxide of hydrogen, mercuric bacteride, and Sulphur Fumigating Candles. The "Sanitas" toilet soaps and powders were shown in considerable variety.

Messrs. Major and Co. (Limited), Sculcoates, Hull, showed their Bath Eucryl, a most pleasant disinfectant for the bath, and their Eucryl toilet soaps, tooth paste, and powder, which merit the favour of the public as first-class toilet preparations.

Messrs. A. Boake Roberts and Co. (Limited), of Stratford, E., had on view their Sulphur Dioxide, and also "Vitrine," a new composition for making cement joints in sanitary work. Vitrine claims to be proof against the action of acids.

Jeyes' Sanitary Compounds Company (Limited), 64, Cannon Street, E.C., showed specimens of their various preparations, including Creolin, Surgical Dusting Powder, and Surgical Soap. Attention was directed to the official report of the senior analyst for Cape Town, in which this firm's products were highly spoken of.

Messrs. Newton, Chambers, and Co. (Limited), of Thorncliffe, near Sheffield, exhibited their Izal Disinfectant and other Izal preparations. The products of this firm have found much favour with the medical profession, and as a household disinfectant Izal is quite safe while being very effective.

The important exhibit of Messrs. J. F. Macfarlan and Co., of Abbeyhill, Edinburgh, included Listerian surgical dressings, antiseptics (double cyanide of mercury and zinc), and also anæsthetics, their specially purified chloroform being a preparation for which this firm is celebrated.

The stall of Messrs. Southall Bros. and Barclay (Limited), of Birmingham, was one that specially interested nurses. Their nurses' bags—medical, surgical, and obstetrical—nurses' wallets and pocket cases for instruments, and chatelaines, claimed much attention. The sanitary towels and accouchement outfits were an important feature of this exhibit.

The "Petanelle" surgical dressings, hygiene toweline, disinfecting powder, bedding, and other "Petanelle" products were largely exhibited by Messrs. Paté, Burke, and Co., 6, Wool Exchange, E.C. These preparations are antiseptic and deodorant in their nature, while the elasticity of the "Petanelle" wool renders it specially serviceable for surgical purposes.

Filters were represented by the Berkefeld Filter Company (Limited), 121, Oxford Street, W., whose pressure filters for hospital and household use were shown in operation; and by Messrs. J. Defries and Sons (Limited), 147, Houndsditch, E.C., who exhibited their Pasteur Filters and also their Equifex Disinfecting Apparatus.

Medical publications were to be found on the stalls of Messrs. J. Bale, Sons, and Danielsson (Limited), 83, Great Titchfield Street, W., who make a speciality of books for dental surgeons. The exhibit of Messrs. Rebman (Limited), 129, Shaftesbury Avenue, W.C., included a medical reference hand atlas, the illustrations being beautiful examples of lithography. The Scientific Press (Limited), of 28 and 29, Southampton Street, Strand, W.C., had a considerable display of their publications on medicine, surgery, and hygiene, and of works dealing with hospitals and nursing. The charts of Messrs. Wodderspoon and Co., 7, Serle Street, W.C., were much in evidence. Other exhibitors were Mrs. A. S. Ballin, 5, Agar Street, W.C.; the London Scottish Engraving Company (Limited), 19, Cursitor Street, E.C.; and the *Medical Times*, 11, Adam Street, W.C, who showed specimens of their various publications.

